# Characteristics of 985 pediatric burn patients in the south of Liaoning province of China

**DOI:** 10.4103/2321-3868.137605

**Published:** 2014-07-28

**Authors:** Hongjun Zhai, Shuangrong Liu, Li Jiang, Bo Sun, Shijie Xin

**Affiliations:** 1 Burn Center, Anshan Hospital of the First Hospital of China Medical University, Anshan, Liaoning, China; 2 Department of General Surgery, The First Hospital of China Medical University, Shenyang 110001, Liaoning, China

**Keywords:** Burns, pediatric, scald

## Abstract

Accidental injury due to burns is a serious and common, but preventable, occurrence in children. To analyze the characteristics of pediatric burns in the south of Liaoning province of China, a retrospective review was conducted of information, including general characteristics, demographics, etiology of burns, anatomical areas burned, and severity of injuries, obtained from medical records of pediatric burn patients admitted to the Burn Center of Anshan Hospital of the First Hospital of China Medical University from 2002 to 2011. Differences between age-groups and cause and severity of injuries were examined using Cochran-Mantel-Haenzsel (C-M-H) statistic or chi-square (χ^2^) analyses where appropriate. A total of 985 pediatric burn cases were included, with only one death. The maximal burn area recorded was 80% and the maximal third-degree burn area was 45%. The majority of burns (637/985, 64.67%) were moderate second-degree wounds, encompassing 5–14% of the total body surface area. The infant age-group (<3 years old) had the largest representation (622/985, 63.15%), with more males than females affected. Most of the injuries occurred at home in children living in the local region. Scalding accounted for 89.85% (885/985) of all injuries, with a decreasing incidence with age, whereas injuries due to flames and from electrical sources markedly increased with age. Only a minority of guardians (244/985, 24.77%) had burn prevention knowledge, and none of them knew how to provide first-aid treatment for burn injuries. These results indicate that the majority of pediatric burns occur in children less than 3 years of age from scalds received while at home. As a large proportion of these cases occurred in rural areas, programs emphasizing burn prevention and treatment knowledge should therefore be made more available to these families.

## Introduction

In China, accidental injury is the first cause of death in children less than 14 years old, with more than 50,000 deaths per year. The second leading cause of these accidental injuries is due to burns,[1] which can result in scar hyperplasia, deformities, functional limitations and growth restriction. Furthermore, the long-term treatment and rehabilitation process can lead to physical, psychological, and economic problems for both the victims and their families. The Burn Center of Anshan Hospital of the First Hospital of China Medical University was established in 1958 with 80 beds for the treatment of patients with acute burns. This Burn Center serves a population of 10 million registered residents of Anshan, and also receives patients from the south of Liaoning province as well as neighboring provinces. The objective of this study was to analyze the epidemiologic characteristics of pediatric burns within this population in order to identify the most vulnerable population that would receive the greatest benefit from burn prevention programs.

**Table:** 

Access this article online	
**Quick Response Code**:	**Website**: www.burnstrauma.com
	**DOI**: 10.4103/2321-3868.137605

## Materials and methods

### Patient selection

A retrospective review was performed on medical records of acute pediatric burn patients (aged 0–14 years) admitted to the Burn Center of Anshan Hospital of the First Hospital of China Medical University between 2002 and 2011. Patients were classified into 3 groups according to their age: An infant group (<3 years old), a kindergarten group (from 3 to <7 years old), and a school-age group (7–14 years old). The severity of burns was classified according to the standards formulated by the Chinese Burn Association into 4 grades: Mild [second-degree wounds to <5% of the total body surface area (TBSA)], moderate (second-degree wounds to 5–14% TBSA or third-degree wounds to <5% TBSA), extensive (second-degree wounds to 15–24% TBSA or third-degree wounds to 5–10% TBSA), and critical (second-degree wounds to ≥25% TBSA or third-degree wounds to ≥10% TBSA). General characteristics, demographic information, etiology and severity of burns, anatomical areas burned, and outcomes were obtained from medical records.

### Statistical analysis

All statistical analyses were conducted using Statistical Analysis System (SAS) statistical software (version 9.1; SAS Institute Inc., Cary, NC, USA). Characteristics of all burns are presented as the number and percentage of cases for categorical variables, with mean ± standard deviation for continuous variables. Cochran-Mantel-Haenszel (C-M-H) statistic or chi-square (χ^2^) tests were used where appropriate for comparisons between categorical variables with a *P* < 0.05 considered as statistically significant.

## Results

### Demographics

A total of 985 acute pediatric burn patients (617 males, 368 females) were admitted to the Burn Center between 2002 and 2011. The average age was 3.12 ± 3.09 years old, ranging from 8 days to 14 years of age. The majority (622/985, 63.15%) of the pediatric burn patients were in the infant group [Table 1]. C-M-H statistic revealed a significant gender difference among the age groups (*P* = 0.0009). The sample population included a total of 599 cases (599/985, 60.81%) from the local population, including 215 urban and 384 rural residents. An additional 343 (343/985, 34.82%) cases were from the south of Liaoning province, and 43 cases (43/985, 4.37%) were from an outside province.

**Table 1:** **Gender distribution by age-group**

Group	Males *n* (%)	Females *n* (%)	Total *n* (%)
Infant	395 (64.02)	227 (61.68)	622 (63.15)
Kindergarten	125 (20.26)	106 (28.80)	231 (23.45)
School age	97 (15.72)	35 (9.51)	132 (13.40)
Total	617 (62.64)	368 (37.36)	985 (100)

Infant group, < 3 year; Kindergarten group, 3–7 year; School-age group, 7–14 year; Cochran-Mantel-Haenszel (C-M-H) statistic = 13.99, *P* = 0.0009

### Etiology of burn

The main cause of burns in this study was from scalding, accounting for 89.85% (885/985) of all pediatric cases, which was not affected by age or gender [Table 2]. The proportion of burns due to scalding dramatically decreased with each increase in age-group, whereas burns from flames, firecrackers, and electrical injuries markedly increased. Other causes of burn injuries were from a stove or hot water pipes. Fourteen of the 18 injuries (77.78%) caused by firecrackers, and 91.67% (11/12) of electrical injuries, occurred in males. Burn injuries due to flames, firecrackers, and electrical causes occurred more frequently in patients from rural areas with corresponding rural to urban ratios of 2.06:1, 2.60:1 and 3.00:1 [Table 3]. Most of the burns to children in the infant and kindergarten age-groups occurred at home (838/853, 98.24%) [Table 4]. However, for children in the school age group, this incidence dropped to 62.88% (83/132). Burns rarely occurred in the kindergarten or at school (10/985, 1.02%).

**Table 2:** **Burn etiology by age-group**

Age-group	Scalding *n* (%)	Flames *n* (%)	Firecrackers *n* (%)	Chemicals *n* (%)	Electrical *n* (%)	Others *n* (%)	Total *n* (%)
Infant	604 (68.25)	5 (9.62)	1 (5.56)	3 (75.00)	0 (0.00)	9 (64.29)	622 (63.15)
Kindergarten	211 (23.84)	10 (19.23)	2 (11.11)	0 (0.00)	5 (41.67)	3 (21.43)	231 (23.45)
School age	70 (7.91)	37 (71.15)	15 (83.33)	1 (25.00)	7 (58.33)	2 (14.29)	132 (13.40)
Total	885 (89.85)	52 (5.28)	18 (1.83)	4 (0.41)	12 (1.22)	14 (1.42)	985 (100)

Infant group, <3 year; Kindergarten group, 3–7 year; School-age group, 7–14 year

**Table 3:** **Burn etiology by residence demographics**

Etiology	Urban *n* (%)	Rural *n* (%)	Total *n* (%)
Flames	17 (68.00)	35(61.40)	52 (63.41)
Firecrackers	5 (20.00)	13(22.81)	18 (21.95)
Electrical	3 (12.00)	9(15.79)	12 (14.63)
Total	25 (30.49)	57 (69.51)	82 (100)

**Table 4:** **Location of injury by age-group**

Age-group	At home *n* (%)	Outdoor *n* (%)	In the kindergarten *n* (%)	At school *n* (%)	Total *n* (%)
Infant	615 (66.78)	3(5.55)	4 (50.00)	0 (0.00)	622(63.15)
Kindergarten	223 (24.21)	4(7.41)	4 (50.00)	0 (0.00)	231(23.45)
School age	83 (9.01)	47(87.04)	0 (0.00)	2 (100)	132(13.40)
Total	921 (93.50)	54(5.50)	8 (0.80)	2 (0.20)	985(100)

Infant group, < 3 year; Kindergarten group, 3–7 year; School-age group, 7–14 year

### Anatomical areas and degree of burns

For this study, burns were categorized into 4 distinct anatomic sites, including head and neck, upper limb, lower limb (including stern) and trunk (including perineum). The head and neck region showed the fewest instances of burns (404/2129, 18.98%)), and the overall majority of burned areas (1885/2129, 88.54%) were second-degree burns [Table 5]. Third-degree burns were more likely to affect the limbs (172/244, 70.49%).

**Table 5:** **Anatomic distribution and severity of burns**

Location	Second degree *n* (%)	Third degree *n* (%)	Total *n* (%)
Head and neck	373(19.79)	31 (12.70)	404 (18.98)
Upper limb	502(26.63)	88 (36.07)	590 (27.71)
Lower limb	484(25.68)	84 (34.43)	568 (26.68)
Trunk	526(27.91)	41 (16.80)	567 (26.63)
Total	1885(88.54)	244 (11.46)	2129 (100)

### Severity and outcome

**Figure 1:**
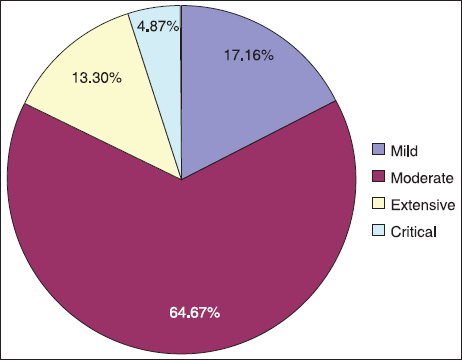
Distribution of burn severity among 985 pediatric patients. Graphical representation showing the proportion of pediatric burns classified as mild [second-degree wounds to <5% of the total body surface area (TBSA)], moderate (second-degree wounds to 5–14% TBSA or third-degree wounds to <5% TBSA), extensive (second-degree wounds to 15–24% TBSA or third-degree wounds to 5–10% TBSA), and critical (second-degree wounds to ≥25% TBSA or third-degree wounds to ≥ 10% TBSA).

Out of the 985 total pediatric cases, there was only one death. The overall average TBSA burned was 9.85 ± 7.22% (range: 1–80%). The maximal full-thickness body surface area was 45%. The majority of cases were moderate burns, which accounted for 64.67% (637/985) of the total [Figure 1]. Burns with TBSA above 25% (critical) accounted for only 4.87% (48/985) of all cases. There were no significant differences in burn severity among the age-groups. No inhalation injury occurred. Seven patients were complicated by septicemia with TBSA from 8% to 30%. All of them recovered.

### Knowledge about burns

Only a minority of patient guardians (244/985, 24.77%) had some knowledge concerning burn prevention. Furthermore, although 45.48% (448/985) of patients had been trained in the kindergarten or at school on how to prevent burns, none of them had any knowledge of first aid treatment following a burn.

## Discussion

Burns are among the most traumatic of injuries and can impose significant psychological, educational, social, and future occupational limitations to a young child.[2] The incidence of hospitalization for pediatric burns is highest in Africa, lowest in the Americas, and with similar rates in Europe, the Middle East and Asia, which bears over half of the world’s pediatric burn cases because of its population size.[3] Between 2002 and 2011, 985 pediatric burn patients were admitted to our Burn Center in Anshan, the majority of which were less than 3 years of age. A large proportion of infants in pediatric burn populations was also reported in an earlier study,[4] indicating that children in this age group are not mature enough to protect themselves, and require greater attention from parents or other guardians.

A greater proportion of the pediatric cases in this study were males, with a male to female ratio of 1.68:1 that is higher than that of other reports.[5] The increased male population observed in the present study may be due to the traditional Chinese preference for sons, especially in rural areas, who serve as the main workforce. According to the China National Bureau of Statistics data reported in 2011, the male to female ratio in the countryside is higher than the overall national ratio. Moreover, the majority of pediatric cases present in our population were local rural residents, despite the fact that only 37.85% of the population of Liaoning province is rural. The higher incidence may be related to the fact that supervision, security and facilities in rural families are generally poorer than those found in urban families.[6] Therefore, prevention programs focused on pediatric burns should be targeted to rural populations.

Results of this study also demonstrate that almost 90% of the pediatric burns were a result of scalding that occurred at home, much higher than an earlier report from the Middle East.[7] Unlike Western countries where hot water temperature is regulated to avoid scalding,[8] in China, hot water stored for cooking, washing and bathing is a primary source for burns to children.[9] As a result, young children who are at home for most of the day are most vulnerable. Burns to children in this study often occurred when they were unsupervised in an area where they could reach the hot water. Thus it is important for the guardians to be aware of the potential hazards in the home, especially at meal and bath times. Furthermore, some habits should be changed in order to prevent burns. For example, bath water for infants is typically prepared by first pouring boiling water into the basin and then adding cold water. To prevent burns from children accidentally falling into the basin, the order should be reversed.[10]

Although the majority of pediatric burns can be avoided by preventing scalding, flame and electrical burns should not be ignored because of the poorer associated prognosis, such as amputation, scar hyperplasia and physical dysfunction. As the age of the patients increased, the proportion of burns due to scalding decreased while flame and electrical injuries markedly increased, which is consistent with findings from a study in India.[11] However, widespread burn prevention knowledge in older children in China is lacking,[12] and thus the most likely causes of burns and prevention strategies for each age group should be emphasized. In addition, the risk of burns from flames, firecrackers and electricity should be stressed in school-aged males in rural areas where the incidence of burns from these sources was highest.

Consistent with findings from a pediatric burn study in the US, the majority of cases brought to the burn clinic were of moderate severity.[13] It is possible, however, that burns of a milder severity did not require hospitalization and were not included in these studies, resulting in an underestimation of the number of mildly burned children. The small proportion of critical burns should not be overlooked, as treatment for these patients involves the greatest medical cost, and long-term physical and psychological dysfunction.

As the majority of pediatric burns are preventable,[14] a greater concerted effort from family members, schools, and the community should be implemented. As infants have no awareness of potential dangers, they are particularly vulnerable to burns from hot fluids placed where they could reach without proper supervision, especially at meal or bath times.[15] The observation in the present study that only one quarter of guardians had some knowledge about burn prevention is consistent with the fact that the majority of burns were scalding injuries to infants. Government agencies, teachers, medical personnel and the media all have the opportunity and responsibility to publicize burn prevention. The media, including newspapers, radio and television, is a particularly effective means and should be exploited for the dissemination of burn prevention knowledge concerning scalding from hot liquids and risks associated with flammable substances and electricity in high-risk environments (home) and populations (infants).[16]

## Burn camp taking roots in China

Twenty-eight smiling, excited kids played with cheers and laughter in the swimming pool at Chunmiao Burn Camp Tuesday, they splashed water and played water polo and had really good time.

Nothing would distinguish these kids from any others who join other camps—except that each of these children is on the recovery trail from different causes of burns or trauma.

Chunmiao Burn Camp started two years ago and this is the third year. It is an effort sponsored by Institute of Burn Research, Southwest Hospital, Chongqing, China, funded by the Chunmiao Charities Aid Foundation for Burned Children, and organized by a Charity group named MSI (Medical Service International). All of the children were one-time patients at the Burn Unit of Institute of Burn Research. Some of the doctors, nurses and rehabilitation therapists at the burn unit take active roles in running the camp.

One of them is Prof. Jun Wu, the Editor-in-chief of Burns & Trauma and the director of Institute of Burn Research. Wu said the camp is a great chance for kids who suffered from similar injuries finds companies and learn coping skills when going back to their own lives.

“This is a place to give burn patients a chance to find new friends,” Yachin Zhou, a rehabilitation therapists for Children and one of the organizers of the camp said. “Kids with burn scars, skin grafts, contractures and even amputations of the extremities may think they are so different from the other children around them — here, they will know that there are still other more children and adults who just suffered from the same experiences as they did.”

Burn camp is recognized as an effective tool all over the world to help burn children recovery after the acute phase of their injury. But, there is no such camp in China before 2012. In contrast, China has the second large population of children under age of 15; burn injury is ranked the fourth place of the accidental injuries in children. Although there is still lack of official data numbered exactly how many children burned each year around China, the fact maybe astonishing. Taking burn unit of Institute of Burn Research for example, burn children can constitute over 50% of outpatients and 30% of inpatients, while the inpatients number of the unit reaches over 1000 during the past years.

As one of the largest and most influential burn centers in China, the Institute of Burn Research now focus more on the quality of life of burn patients and dedicates to the integration of physical and occupational therapies with clinical treatments, “our goal is to give burn patients dignified lives rather than just wound healing.” Prof. Wu said.

With the help of volunteer organizations and companies like Chunmiao Charities Aid Foundation for Burned Children, MSI (Medical Service International), Summer Camp Odeman, Anhui Anke biotechnology (Group) CO. LTD, the Chunmiao Burn Camp has run successfully for the third year. It not only provides a place for these kids to find and make friends, but also a chance for medical staffers to see the other side of their journey.

More information on Chunmiao Burn Camp can be found through the Web site at www.burncamp-china.com.
